# Supplementary material for: CD206+ M2-like macrophages regulate systemic glucose metabolism by inhibiting proliferation of adipocyte progenitors
Source: Nat Commun. 2017 Aug 18;8:286. doi: 10.1038/s41467-017-00231-1 (PMC5561263; doi:10.1038/s41467-017-00231-1)
Supplement: Supplementary file 2 — Supplementary Information [file 41467_2017_231_MOESM2_ESM.pdf]

Title of file for HTML: Peer Review File
Description:

Title of file for HTML: Supplementary Information
Description: Supplementary Figures and Supplementary Table

Supplementary Figure 1

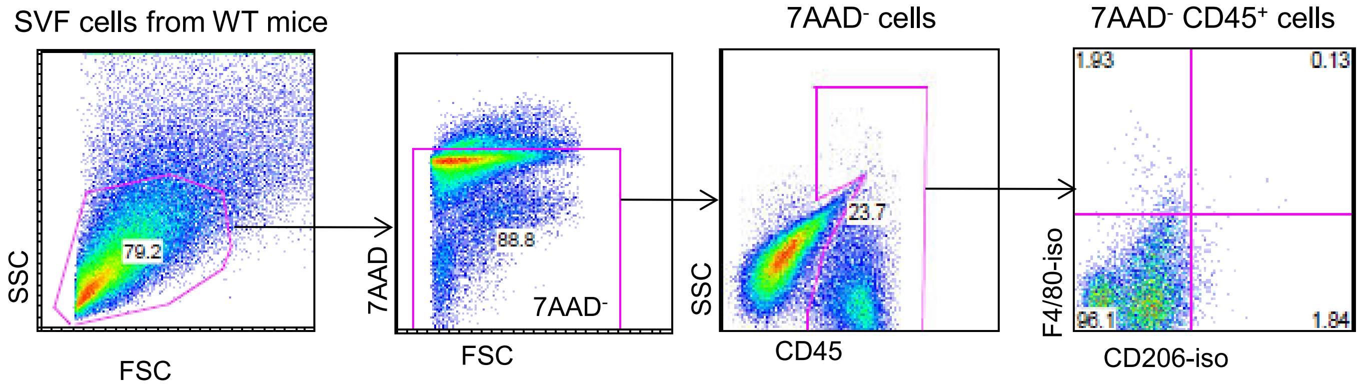

Supplementary Figure 1

Gating strategy for F4/80$^+$CD206$^+$ M2-like macrophages in adipose tissue

Representative flow cytometry analysis of the eWAT. Cells in the SVF of eWAT from C57BL/6 mice were analyzed using flow cytometry. Cells were isolated from enzymatically digested mouse eWAT. After the exclusion of doublets and debris, dead cells were excluded by 7AAD staining. Leukocytes in SVF were identified by CD45 staining. To identify F4/80 and CD206 expression, CD45$^+$ cells were stained with isotype control antibodies for anti-F4/80 and anti-CD206 antibodies

Supplementary Figure 2

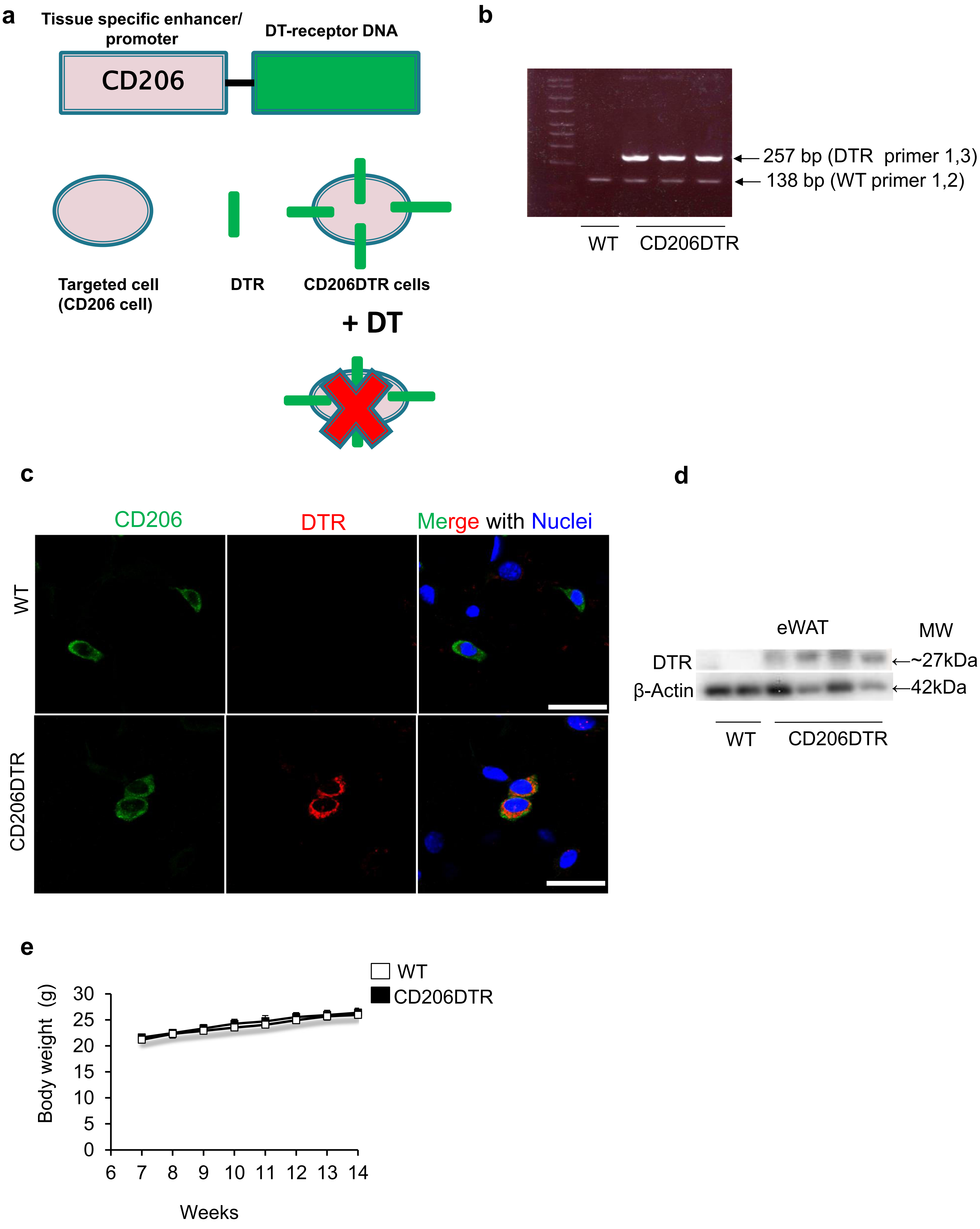

Supplementary Figure 2
Creation of CD206DTR mice

(a) Schematic representation of creation CD206DTR mice. Depletion of CD206 M2-like macrophages by DT administration. **(b)** PCR-genotyping analysis showing a 257-bp specific DTR sequence in CD206DTR mice. **(c)** Confocal immunofluorescence double staining of DTR and CD206 in eWAT. The images were taken by using confocal miscroscope Leica TCS-SP5, 63x oil, scale bar, 25 μm. **(d)** Western blot analysis showing DTR protein levels in the eWAT of CD206DTR mice. **(e)** Body weight changes of CD206DTR mice and WT littermates (n=3-5 mice per group). The data are shown as the means ± SEM. $^*P < 0.05$ compared with littermates by Student's t-test.

Supplementary Figure 3

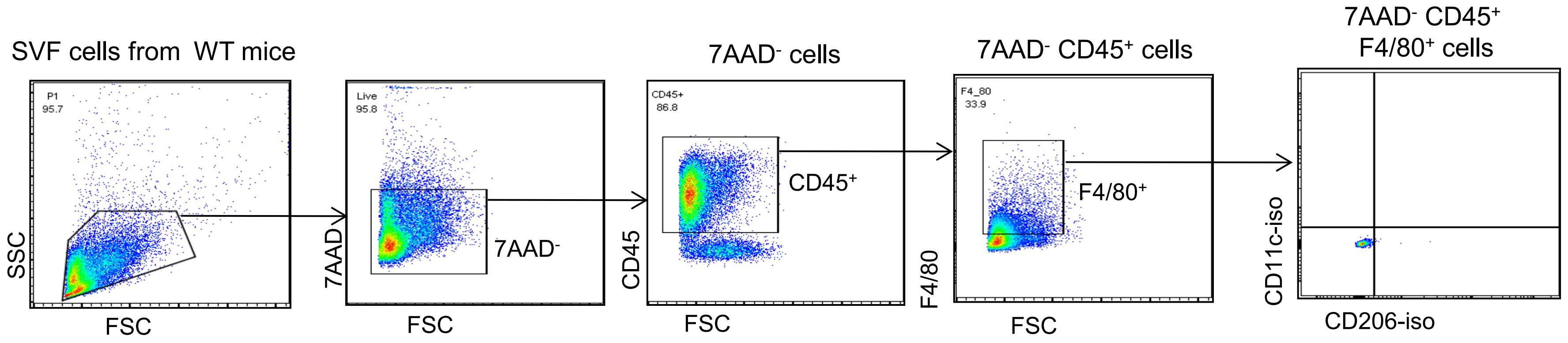

Supplementary Figure 3
Gating strategy for M1- and M2-like macrophages

Cells were isolated from enzymatically digested C57BL/6 (or WT) mouse eWAT. After the exclusion of doublets and debris, dead cells ($7AAD^+$ cells) were excluded by 7AAD staining. Leukocytes in SVF were identified by CD45 staining. $CD45^+$ cells were further gated for F4/80-positive macrophages. To identify M1 and M2 macrophages, $CD45^+F4/80^+$cells were stained for CD11c and CD206 respectively.

Supplementary Figure 4

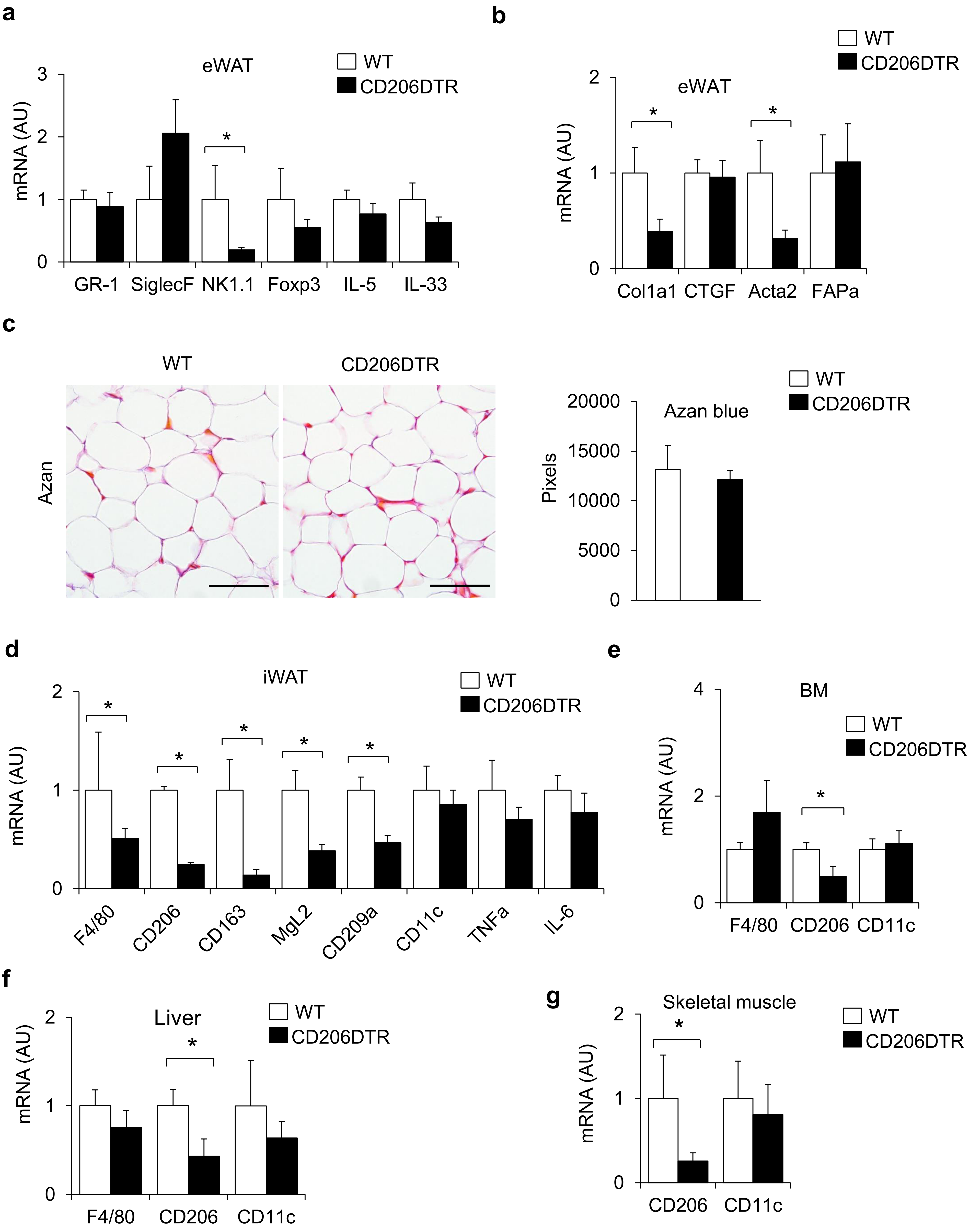

Supplementary Figure 4

Gene expression analysis of WAT and BM

(a) The relative mRNA expression of various hematopoitic cell marker genes in the eWAT. **(b)** The relative mRNA expression of fibrosis related marker genes in eWAT. **(c)** Representative images of Azan-stained sections of eWAT from DT-treated WT and CD206DTR mice. Scale bars, 500 µm. Fibrotic area (blue) was analyzed by ImageJ software. Data are representative of at least two independent experiments. **(d)** The relative mRNA expression of M1-like and M2-like macrophages markers in the iWAT. **(e, f)** The relative mRNA expression of M1/M2 macrophage markers in the BM and liver and skeletal muscle after DT treatment (n=5-6 mice per group). The data are shown as the means ± SEM. $*P < 0.05$ compared with littermates by Student's t-test.

Supplementary Figure 5

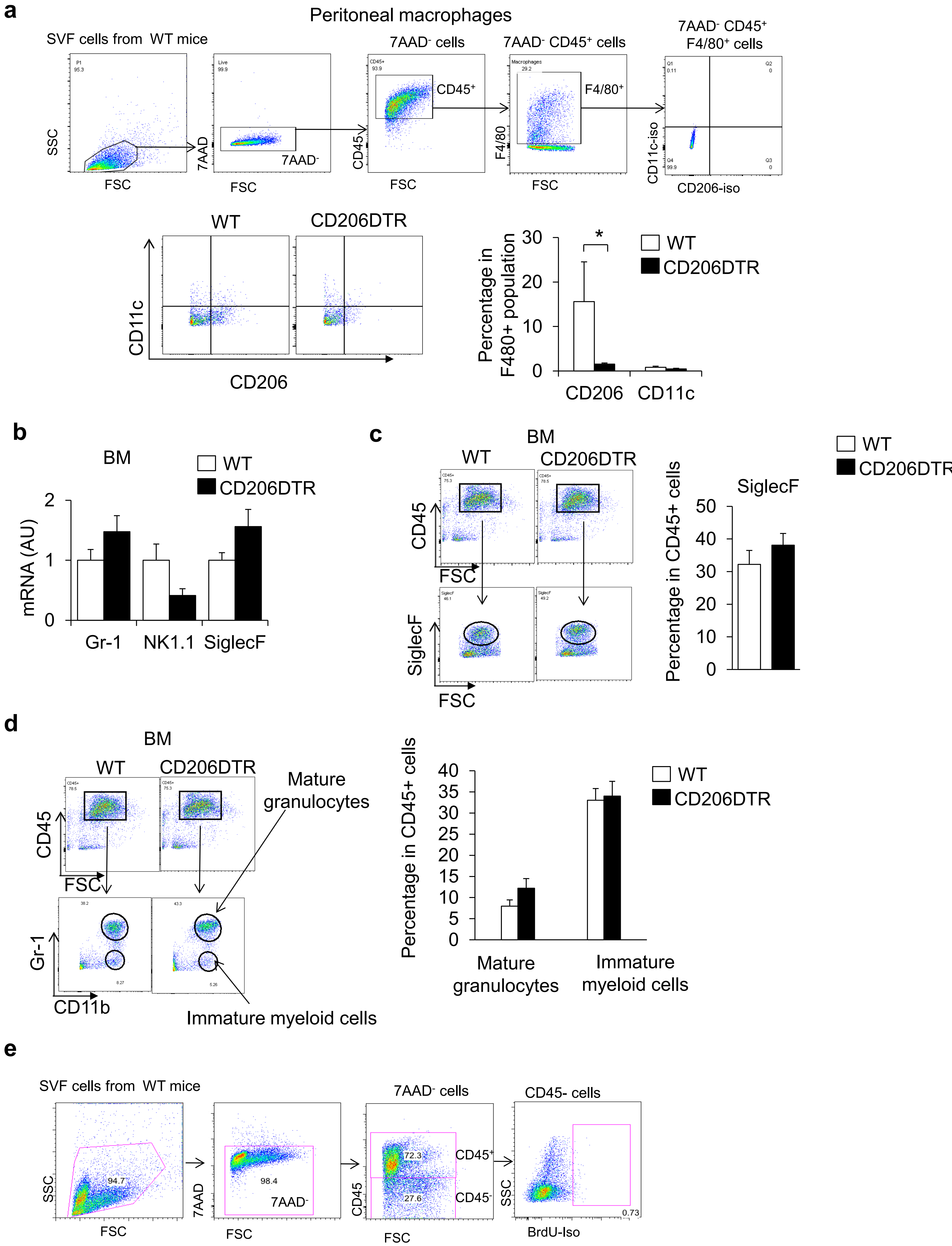

Supplementary Figure 5

Flow cytometry analysis of Peritoneal cavity macrophages and BM

(a) Representative flow cytometry analysis of peritoneal cavity macrophages from WT mice (upper panel) and M1- and M2-like macrophage quantification in WT and CD206DTR mice (lower panel). After the exclusion of doublets and debris, dead cells were excluded by 7AAD staining. Leukocytes in peritoneal cavity were identified by CD45 staining. $CD45^+$ cells were further gated for F4/80-positive macrophages to identify M1 and M2 macrophages. Isotype control antibody for the anti-CD11c and anti-CD206 were used as a negative control. The data are shown as the means ± SEM. $^*P < 0.05$ compared with littermates by Student's t-test.

(b) The relative mRNA expression of granulocytes, natural killer cells and eosinophils in BM from DT-treated WT and CD206DTR mice. The data are shown as the means ± SEM. $^*P < 0.05$ compared with littermate by Student's t-test. **(c)** Representative flow cytometry analysis of eosinophils ($SiglecF^+$) in BM from WT and CD206DTR mice (left panel) and quantification of these cells (right panel). Live ($7AAD^-$) cells were gated for $CD45^+$ cells and then SiglecF fraction was analyzed in $CD45^+$ cells. Isotype control antibody was used as a negative control. The data are shown as the means ± SEM. $^*P < 0.05$ compared with littermates by Student's t-test.

(d) Representative flow cytometry analysis of CD11b and Gr-1 expression in BM (left panel) and quantification of $CD11^{hi}$ $Gr\text{-}1^{hi}$ and $CD11b^{int}$ $Gr\text{-}1^{int}$ cells (right panel). Live ($7AAD^-$) cells were gated for $CD45^+$ cells and then CD11b and Gr-1 expression was analyzed in $CD45^+$ cells. Isotype control antibody for the relative antibodies were used as a negative control. The data are shown as the means ± SEM. $^*P < 0.05$ compared with littermate by Student's t-test. **(e)** BrdU gating; Live SVF cells ($7AAD^-$ cells) from WT mice were gated for $CD45^+$ and $CD45^-$ population. Then BrdU was analyzed in both fraction including $CD45^+$ $BrdU^+$ and $CD45^-$ $BrdU^+$ population. Isotype control antibody for the anti-BrdU was used as a negative control.

Supplementary Figure 6

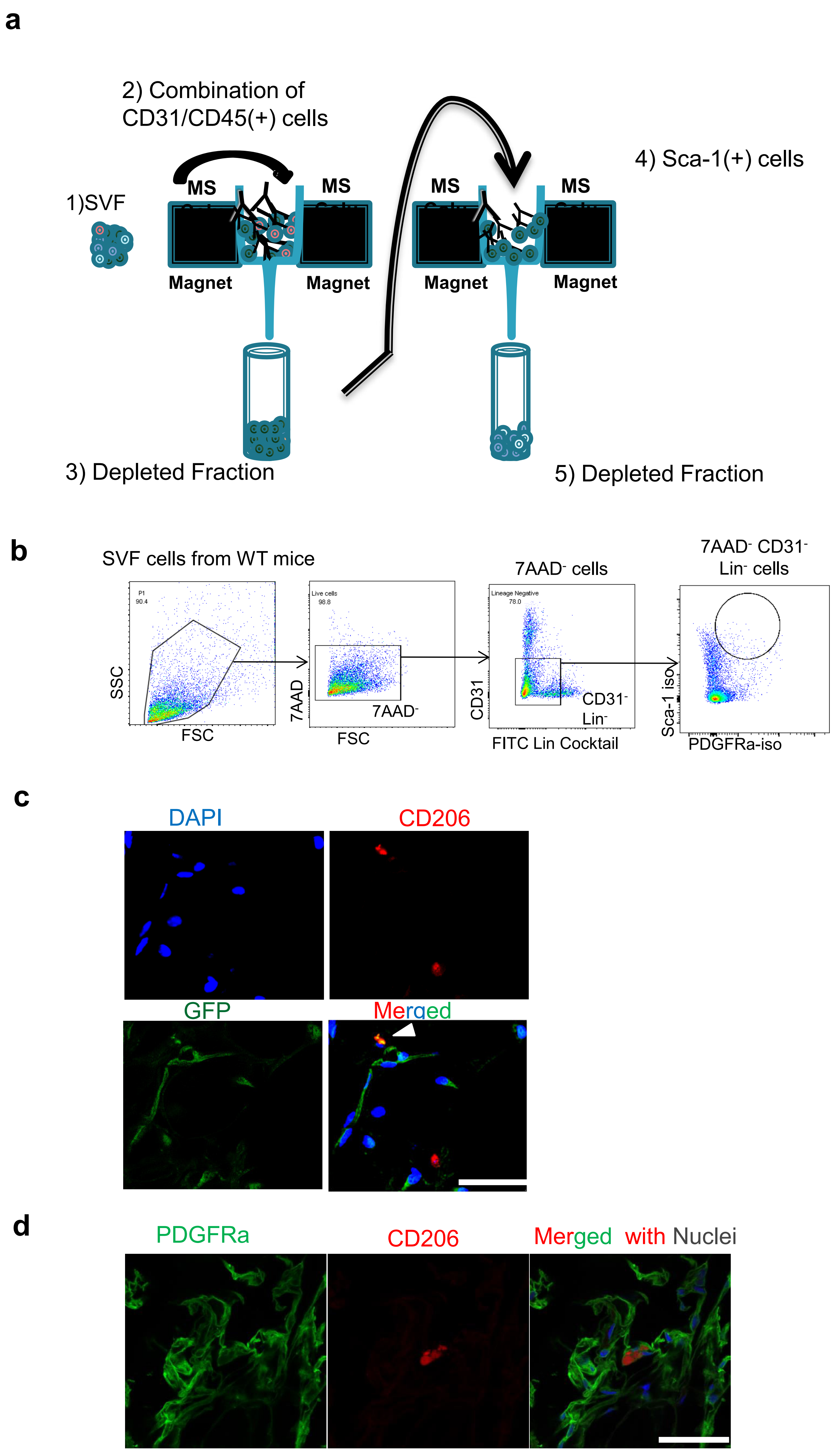

Supplementary Figure 6

Schematic diagram of MACS study and co-localization of CD206 with PDGFRa

(a) A schematic diagram showing the MACS processing. First, the eWAT was fractionated into adipocytes and SVF. The SVF were then processed MACS bead, as described in the methodology section. **(b)** The flow cytometry detection of the APs in SVF from WT mice First, negative selection of $CD31^{+}$ (endothelial), FITC-lineage cocktail cells were selected followed by positive selection of $PDGFRa^{+}$ and $Sca\text{-}1^{+}$ cells (APs). Isotype of antibodies were used as negative control. This experiment was performed with a FACSDiva Version 6.1.2 automated cell analyzer (Becton Dickinson FACSCanto II) and cell sorting was performed by an automatic cell sorting analyzer (Becton Dickinson FACSAria SORP). **(c)** High resolution confocal imaging of frozen eWAT sections from PDGFRa-CreERT2-Egfp (PRa) mice stained for CD206 vs GFP. These pictures were taken by using Leica TCS-SP5 (Magnification 63x) oil. Scale bar, 25 μm. **(d)** Confocal immunofluoresence staining of CD206 co-localized with PDGFRa. These pictures were taken by using LSM780 (Magnification 40x). Scale bar, 25 μm.

Supplementary Figure 7

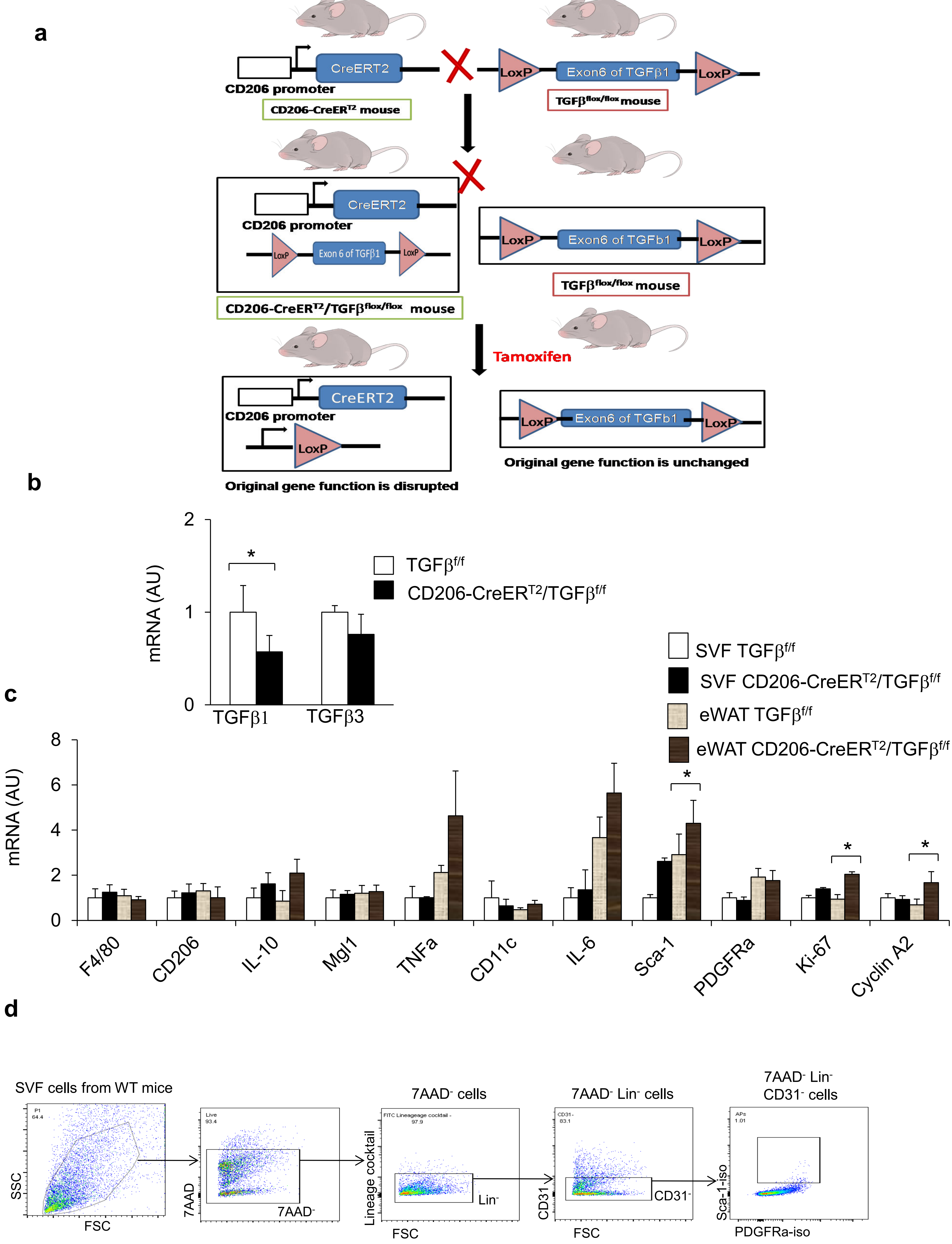

Supplementary Figure 7
Creation of CD206-CreER^{T2}/TGFβ$^{flox/flox}$ mice

(a) A schematic diagram showing creation of CD206-CreER^{T2}/TGFβ$^{flox/flox}$ mice. **(b)** Relative mRNA expression of FACS-sorted CD206^{+} cells from the eWAT of tamoxifen treated CD206-CreER^{T2}/TGFβ$^{flox/flox}$ mice compared with control TGFβ$^{flox/flox}$ mice (n=3). The data are shown as the means ± SEM. **P* < 0.05 compared with littermate by Student's t-test. **(c)** Relative mRNA expression of eWAT and SVF from the eWAT of CD206-CreER^{T2}/TGFβ$^{flox/flox}$ mice compared with control TGFβ$^{flox/flox}$ mice (n=4-5). The data are shown as the means ± SEM. **P* < 0.05 compared with littermates by Student's t-test.
(d) The flow cytometry detection of the APs in SVF from WT mice. First, negative selections of CD31+ (endothelial), FITC-lineage cocktail cells were selected followed by positive selection of PDGFRa^{+} and Sca-1^{+} cells. This experiment was performed with a FACSDiva Version 6.1.2 automated cell analyzer (Becton Dickinson FACSCanto II) and cell sorting was performed by an automatic cell sorting analyzer (Becton Dickinson FACSAria SORP).

Supplementary Figure 8

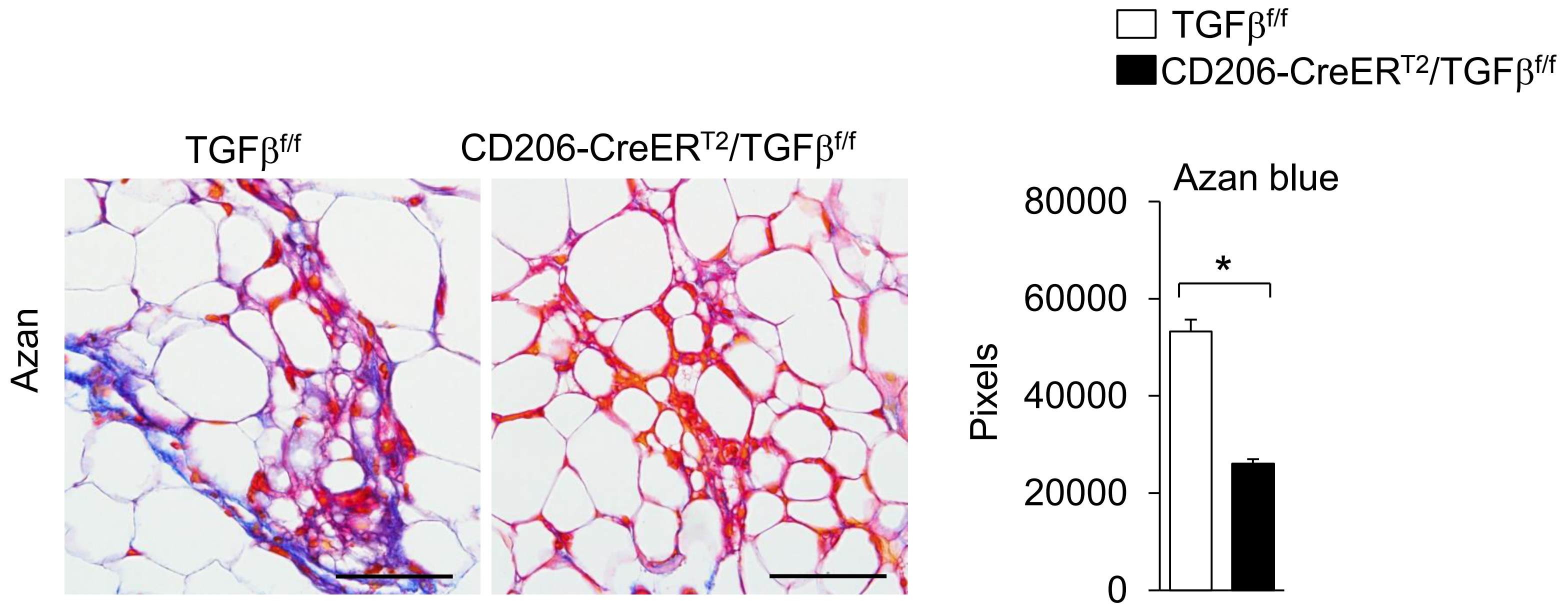

Supplementary Figure 8
Fibrosis of eWAT

Representative images of Azan-stained sections of eWAT from mice used tamoxifen treated CD206-CreER^{T2}/TGFβ1$^{flox/flox}$ mice compared to tamoxifen treated TGFβ1$^{flox/flox}$ control mice. Scale bars, 500 μm. Fibrotic area (blue) was analyzed by ImageJ software. The data are shown as the means ± SEM. $^{*}P < 0.05$ compared with littermates by Student's t-test. Data are representative of at least two independent experiments.

Supplementary Figure 9

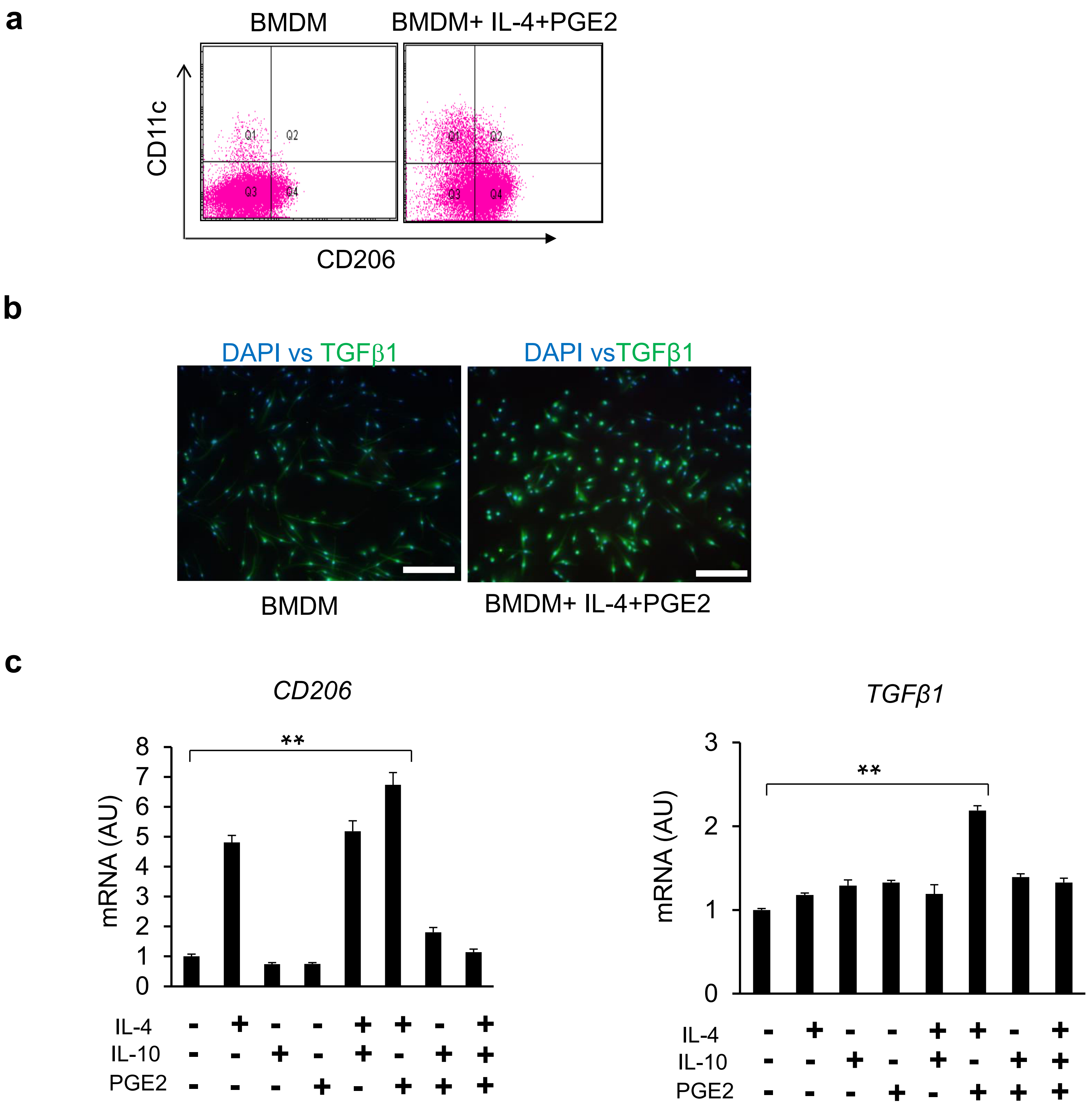

Supplementary Fig. 9

IL-4-induced BMDM expression of *CD206* and TGFβ1

(a) Representative flow cytometry analysis of BMDMs cultured with IL-4 (10 ng/mL, final concentration) and PGE2 (50 ng/mL, final concentration) for 24 hours. **(b)** Immunostaining of CD206 and *TGFβ1*. Scale bar, 100μm **(c)** Relative mRNA expression of *CD206* (upper panel) and *TGFβ1* (lower panel). The data were calculated from experiments with 3-6 dishes per group. The data are shown as the means ± SEM. *P < 0.05, **P < 0.01 (ANOVAs with the post Tukey-Kramer test and Bonferroni correction).

Supplementary Figure 10

a

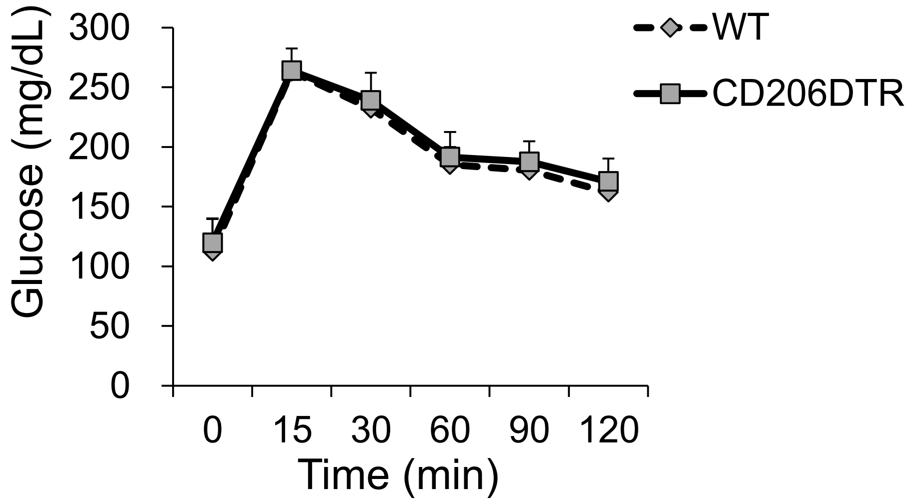

b

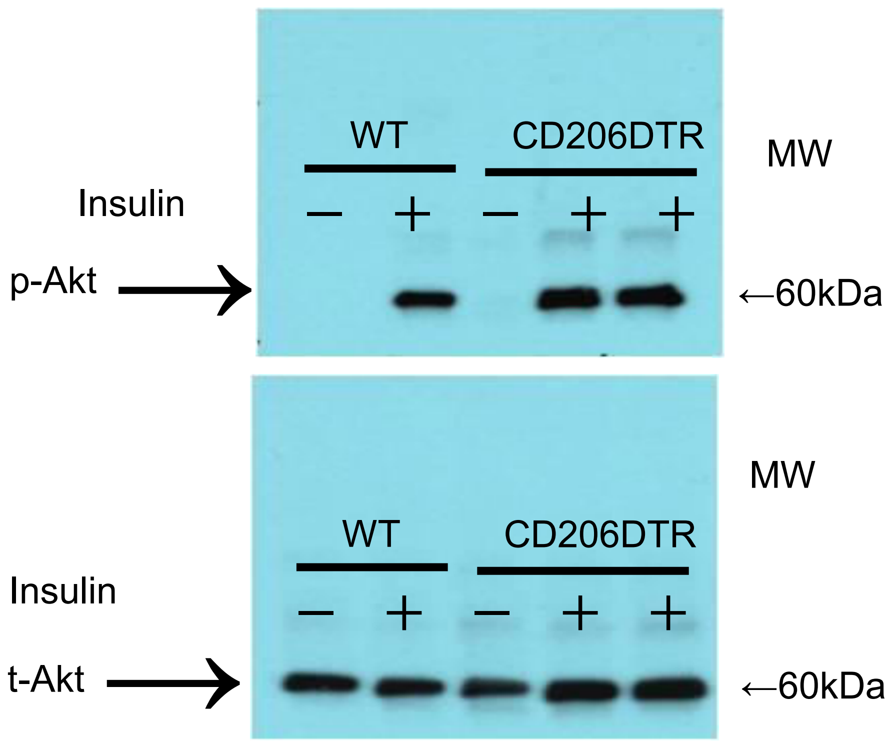

c

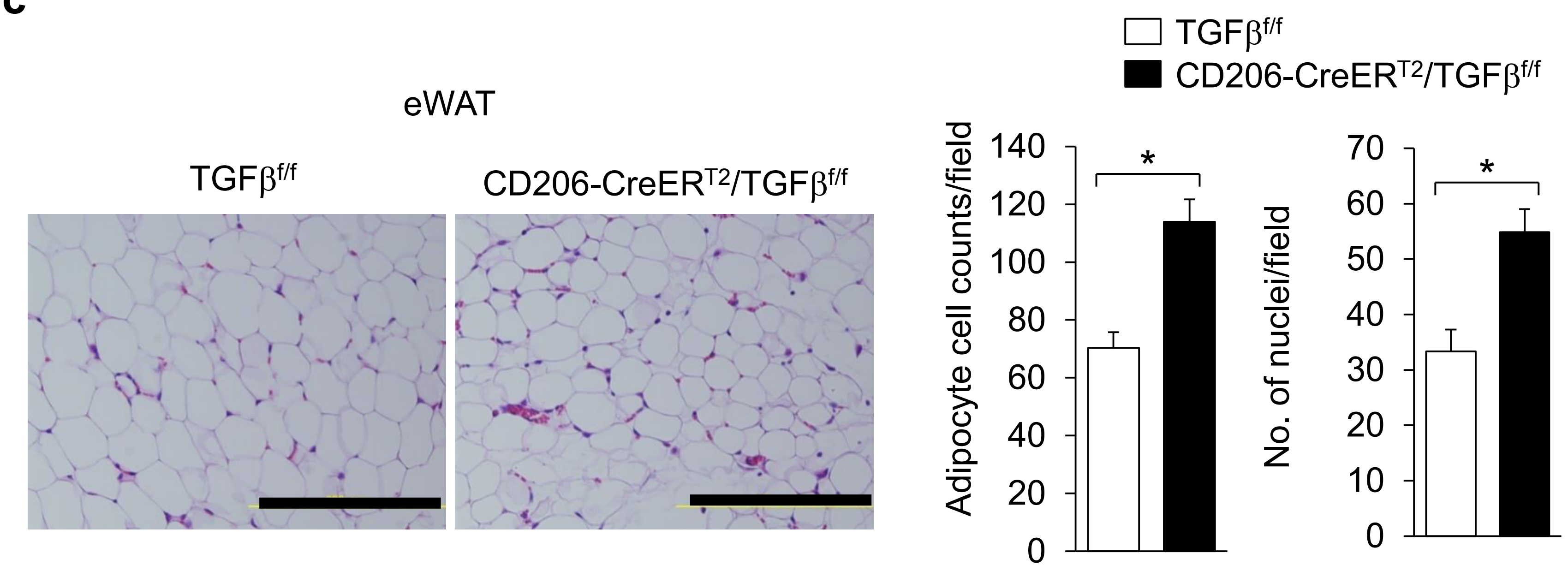

Supplementary Fig. 10

IP-GTT in NC-fed mice and generation of smaller adipocytes

(a) The glucose concentrations in an intra-peritoneal glucose tolerance test (IP-GTT) in NC-fed CD206DTR and WT mice without DT treatment (n=3-5 per group). **(b)** Uncropped scan of western blot of liver from DT-treated WT and CD206DTR mice. **(c)** Representative HE-immunohistochemistry micrographs (left panel) and adipocyte size and cell count measurements (right panel) in the eWAT of CD206-CreERT2/TGFβ$^{flox/flox}$ compared with TGFβ$^{flox/flox}$ control mice are shown. (200x magnifications); Red scale: 200 μm. (n=3 per group). The data are shown as the means ± SEM. **P* < 0.05 compared with littermates by Student's t-test.

Supplementary Figure 11

a

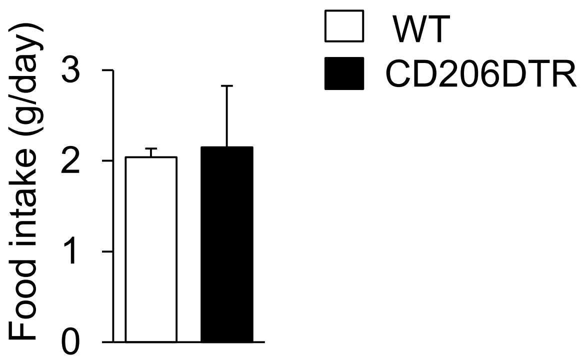

b

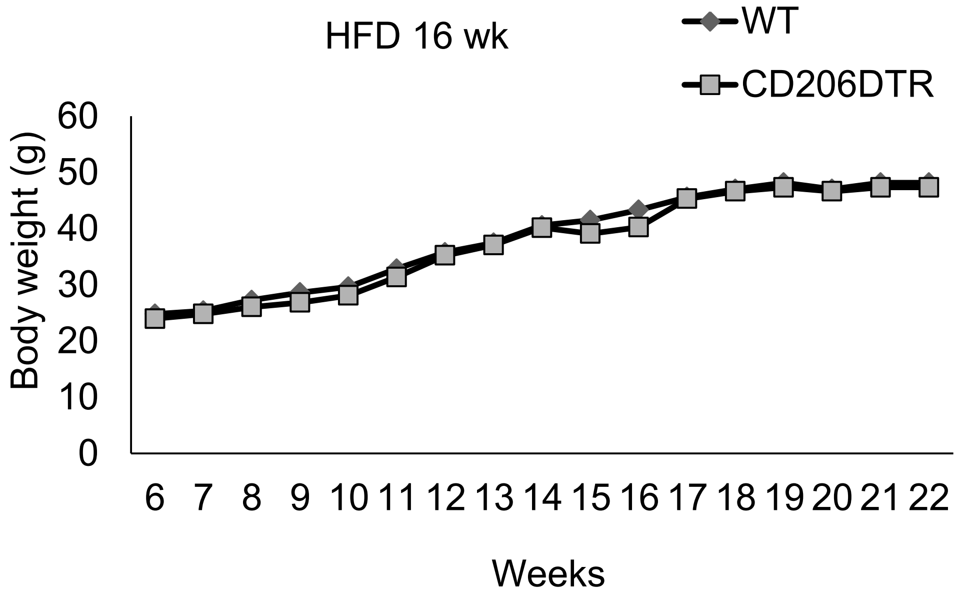

c

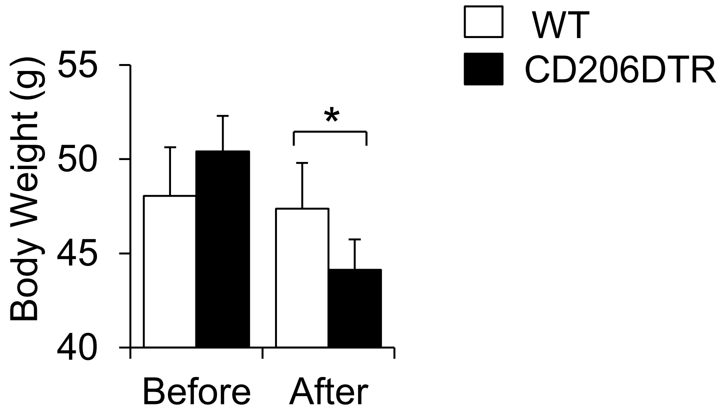

Supplementary Figure 11
HFD mice food intake, body weight, and histogram of the eWAT

(a) Food intake before and after DT-treated HFD-fed CD206DTR and WT mice. **(b)** Body weights of the WT and CD206DTR mice during 16 weeks of HFD (n=4-6 mice per group). **(c)** Body weights before and after DT administration in the HFD-fed CD206DTR and WT mice (n=4-6 mice per group). The data are shown as the means ± SEM. *P < 0.05 compared with littermates by Student's t-test.

Supplementary Figure 13

a

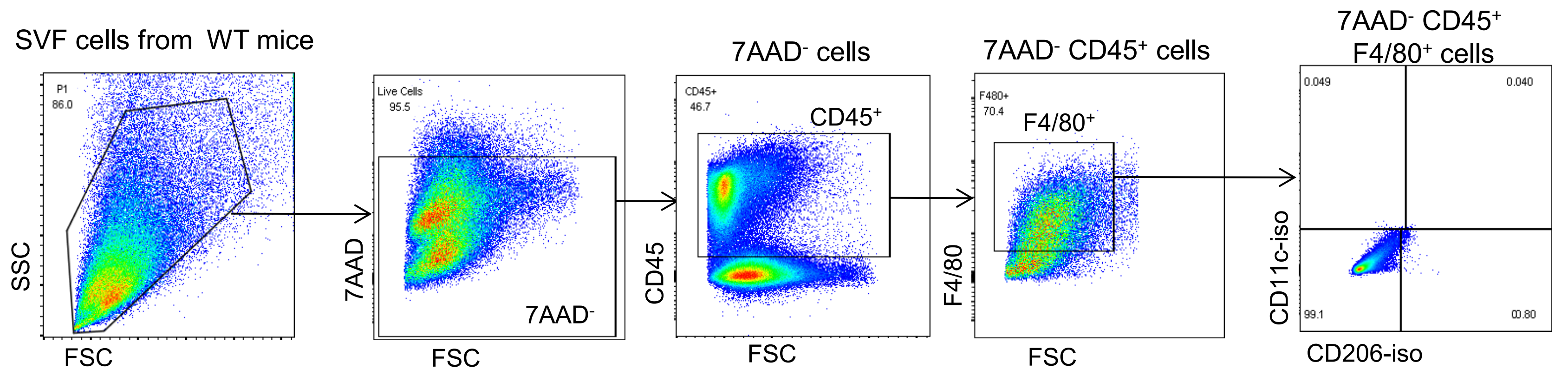

b

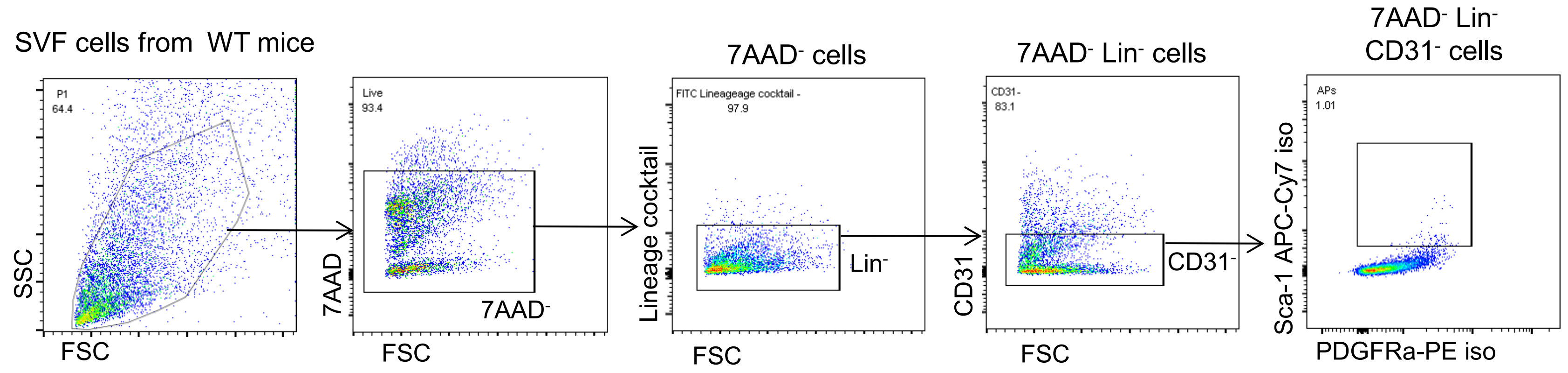

Supplementary Figure 12

Gating strategy for M1- and M2-like macrophages and APs

(a) Gating strategy for M1 and M2 macrophages in SVF. $CD45^+$ $F4/80^+$ $CD11c^+$ population was selected for M1-like while $CD45^+$ $F4/80^+$ $CD206^+$ population was analyzed for M2-like macrophages. FMO of CD206 and CD11c was used to justify the gating. Isotype control antibodies for anti-CD11c and anti- CD206 antibodies were used as negative controls. **(b)** Representative images of flow cytometry detection of the APs in the eWAT. First, negative selection of $CD31^+$ (endothelial), FITC Lineage cocktail cells was selected followed by positive selection of $PDGFRa^+$ and $Sca\text{-}1^+$ cells. These experiments were performed with a FACSDiva Version 6.1.2 automated cell analyzer (Becton Dickinson FACSCanto II).

Supplementary Table 1

The sequences of SYBR Green primers used for RT PCR

Ki-67	Forward: CGCCAACCAAGAGGAAGTCT<br>Reverse: GGGGCCGTTCCTTGATGATT
p15	Forward: CCACCCTTACCAGACCTGTG<br>Reverse: AGGCGTCACACACATCCAG
p16	Forward: GAACTCTTTCGGTCGTACCC<br>Reverse: CGAATCTGCACCGTAGTTGA
p21	Forward: GACATCTCAGGGCCGAAAAC<br>Reverse: CGGCGCTTGGAGTGATAGAA
p27	Forward: AGGAGAGCCAGGATGTCAGC<br>Reverse: CAGAGTTTGCCTGAGACCCAA
p57	Forward: GGAGCAGGACGAGAATCAAG<br>Reverse: GAAGAAGTCGTTCGCATTGG
TGFβ1	Forward: AAGTTGGCATGGTAGCCCTT<br>Reverse: GCCCTGGATACCAACTATTGC
TGFβ2	Forward: AGGAGGTTTATAAAATCGACATGC<br>Reverse: TAGAAAGTGGGCGGGATG
TGFβ3	Forward: CCCTGGACACCAATTACTGC<br>Reverse: TCAATATAAAGGGGGCGTACA
C/EBPα	Forward: TGGACAAGAACAGCAACGAG<br>Reverse: TCACTGGTCAACTCCAGCAC
CD24	Forward: TGCTCCTACCCACGCAGATT<br>Reverse: GCGTTACTTGGATTTGGGGAAGCA
Pref-1	Forward: AACCATGGCAGTGCATCTG<br>Reverse: GCATTCGTACTGGCCTTTCT
Zbtb46	Forward: AGAGAGCACATGAAGCGACA<br>Reverse: CTGGCTGCAGACATGAACAC
Sca1	Forward: TCTGAGGATGGACACTTCTC<br>Reverse: CTCAGGCTGAACAGAAGCAC
